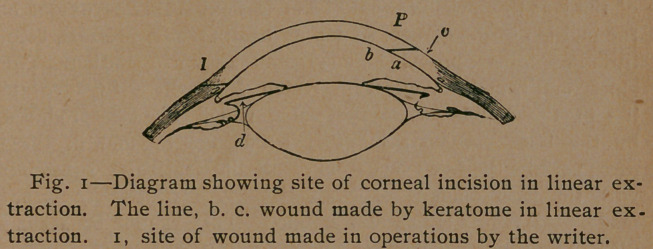# Treatment of Traumatic Cataract

**Published:** 1895-03

**Authors:** James Moores Ball

**Affiliations:** Professor of Ophthalmology and Otology in the St. Louis College of Physicians and Surgeons; President of the Tri-State Medical Society, of Iowa, Illinois and Missouri; Editor of the Tri-State Medical Journal; Member of the Medico-Legal Society of New York, Etc.; 810 Olive street; St. Louis, Mo.


For Texas Medical Journal.

### TREATMENT OF TRAUMATIC CATARACT—

### By Extraction of the Lens.

BY JAMES MOORES BALL, M. D.,
Professor of Ophthalmology and Otology in the St. Louis College of Physicians and Surgeons; President of the Tri-State Medical Society, of Iowa, Illinois and Missouri; Editor of the Tri-State Medical Journal; Member of the Medico-Legal Society of New York, etc.

[Abstract of a paper read before the Mississippi Valley Medical Association, at Hot Springs, Arkansas, November 23, 1894.]

CASES of lenticular opacity, caused by a foreign body which remains in the eye are always extremely dangerous owing to the introduction of pyogenic germs on the one hand, and the dangers of sympathetic ophthalmia on the other. Such cases must always remain an opprobrium to ophthalmology. However there are many cases of traumatic cataract attended by rapid increase of intra-ocular tension, peri-corneal injection, iritis or irido-cyclitis and ultimate excavation of the optic nerve head, in which the foreign body either lodges in the lens or is withdrawn at the time of injury. It is concerning such cases that I wish to speak.

For years the practice of the profession has been to use atropia if the symptom be not severe, and to perform linear extraction if the symptoms be acute. This operation—linear extraction—is a relic of that surgical age when antisepsis was unknown. Performed for the purpose of relieving undue tension and evacuating the lenticular fragments, the very nature of the operation has been such as to diminish the first only temporarily and defeat the second indication frequently. The situation of the corneal incision has been such as to preclude the possibility of removing all the fragments of the swollen lens. The oblique course of the wound has rendered its patency impossible while favoring its closure. Furthermore the incision made by the ordinary keratome was too short. That such objections are not chimerical can be seen by a study of the accompanying diagram:

Fig. 1—Diagram showing site of corneal incision in linear extraction. The line, b. c. wound made by keratome in linear extraction. 1, site of wound made in operations by the writer.

In the cornea we find that flap wounds gape more than linear ones, but the tendency towards gaping depends more upon whether the wound traverses the cornea perpendicularly or obliquely. The former is more particularly the case in wounds made with the Graefe knife in which the knife passes through the cornea from within outwards, while the latter condition exists when the lance knife is used. These wounds do not gape, because the instrument passes obliquely through the cornea and the lips of the wound close like a valve. The closure is caused by intra-ocular pressure. This force presses as strongly upon the posterior lip (*a*) of the wound as upon the anterior (*b*). The wound must be made to gape before the softened lens matter can be evacuated. Gaping of the wound can be produced, not by the application of force opposite the site of corneal incision, but only by pressure applied just peripheral to the wound (at *c* in figure 1). You can readily imagine that such a wound will not permit the removal of all the diseased mass; in fact, only the softest portion of the lens can be evacuated, and irrigation of the anterior chamber is not to be thought of. Such, then, are the objections to an operation which has the sanction of authority and the prestige of age. For reasons already mentioned I contend that this operation is unscientific.

Have I anything better to offer? The proposition which I have to advance is this: In cases of traumatic cataract, with rapid increase of intra-ocular tension, an operation should be performed and that operation should be not linear extraction, as has been the rule heretofore, but an extraction made with the Graefe knife and with the incision located in the corneo-scleral junction. The knife should cut from one-third to two-fifths of the corneal-circumferance, according to the extent to which the softening process in the lens has advanced. If glaucomatous symptoms supervene with softening of only a small part of the lens, the corneal incision should be large, if the softening involves the whole of the lens the incision should be of less extent. The extent of the incision in the cornea, so far as healing is concerned, is of little importance, provided we make an aseptic operation. The chief merit of the operation which I am here to advocate lies in the avoidance of the valve which we saw produced by the linear method; in other words, my method in these cases permits the free evacuation of all the lenticular substance with the least amount of traumatism. An iridectomy is not made. All debris is removed at once. This cannot be accomplished by the linear method.

810 Olive street.

## Figures and Tables

**Fig. 1. f1:**